# Health equity and COVID-19: global perspectives

**DOI:** 10.1186/s12939-020-01218-z

**Published:** 2020-06-26

**Authors:** Efrat Shadmi, Yingyao Chen, Inês Dourado, Inbal Faran-Perach, John Furler, Peter Hangoma, Piya Hanvoravongchai, Claudia Obando, Varduhi Petrosyan, Krishna D. Rao, Ana Lorena Ruano, Leiyu Shi, Luis Eugenio de Souza, Sivan Spitzer-Shohat, Elizabeth Sturgiss, Rapeepong Suphanchaimat, Manuela Villar Uribe, Sara Willems

**Affiliations:** 1 The Cheryl Spencer Department of Nursing, Faculty of Social Welfare and Health Sciences, University of Haifa, 31905 Mount Carmel, Israel; 2 School of Public Health, Fudan University, Shanghai, PR China; 3 Health Collective Institute, Federal University of Bahia, Salvador, Brazil; 4 The mobile clinic for minimizing prostitution damages, Ministry of Health, Haifa, Israel; 5 ”Ve’ahavta” clinic, for refugees and non-citizenship people, Nesher, Israel; 6 Department of General Practice, Faculty of Medicine, Dentistry and Health Science, The University of Melbourne, Melbourne, Australia; 7 Department of Health Policy and Management, School of Public Health, University of Zambia, Lusaka, Zambia; 8 Chr. Michelsen Institute, Bergen, Norway; 9 Bergen Centre for Ethics in Priority Setting (BCEP), University of Bergen, Bergen, Norway; 10 Faculty of Medicine, Chulalongkorn University, Bangkok, Thailand; 11 National Health Foundation, Bangkok, Thailand; 12 The Equity Initiative, CMB Foundation, Bangkok, Thailand; 13 Department of Clinical, Umeå University, Umeå, Sweden; 14 Gerald and Patricia Turpanjian School of Public Health, American University of Armenia, Yerevan, Armenia; 15 Department of International Health, Johns Hopkins University, Baltimore, USA; 16 Center for the Study of Equity and Governance in Health Systems, CEGSS, Guatemala City, Guatemala; 17 Center for International Health, University of Bergen, Bergen, Norway; 18 Health Policy and Management, Bloomberg School of Public Health, Johns Hopkins University, Baltimore, USA; 19 The Azrieli Faculty of Medicine, Bar-Ilan University, Zefat, Israel; 20 Department of General Practice, Monash University, Melbourne, Australia; 21 International Health Policy Program, Ministry of Public Health, Nonthaburi, Thailand; 22 Division of Epidemiology, Department of Disease Control, Ministry of Public Health, Bangkok, Thailand; 23 Bloomberg School of Public Health, Johns Hopkins University, Baltimore, USA; 24 Faculty of Medicine and Health Sciences, Department of Public Health and Primary Care, Ghent University, Ghent, Belgium

**Keywords:** Health equity, Humanitarian crisis, Pandemic, Covid-19

## Abstract

The COVID-19 is disproportionally affecting the poor, minorities and a broad range of vulnerable populations, due to its inequitable spread in areas of dense population and limited mitigation capacity due to high prevalence of chronic conditions or poor access to high quality public health and medical care. Moreover, the collateral effects of the pandemic due to the global economic downturn, and social isolation and movement restriction measures, are unequally affecting those in the lowest power strata of societies. To address the challenges to health equity and describe some of the approaches taken by governments and local organizations, we have compiled 13 country case studies from various regions around the world: China, Brazil, Thailand, Sub Saharan Africa, Nicaragua, Armenia, India, Guatemala, United States of America (USA), Israel, Australia, Colombia, and Belgium. This compilation is by no-means representative or all inclusive, and we encourage researchers to continue advancing global knowledge on COVID-19 health equity related issues, through rigorous research and generation of a strong evidence base of new empirical studies in this field.

## Introduction

With the continuous rise in the global death toll from COVID-19 there is a growing realization that mortality for the SARS-COV-2 virus is inequitably distributed among vulnerable populations. These vulnerable populations may include older adults, people living in densely populated areas, people with lower socioeconomic status, migrants and minorities. These groups have higher rates of comorbid chronic conditions, putting them at high-risk for infection and for severe consequences of the disease [1, 2]. People holding essential roles, usually from lower-paying jobs, such as public transportation operators or grocery store or pharmacy clerks, are more exposed to the public and thus also to being infected. Minorities and migrant populations also face language and cultural barriers limiting their access to accurate information on prevention and mitigation, compelling them to rely on social media to obtain advice that may be erroneous. People with poor access to healthcare who experience COVID-19 related symptoms may delay or even forgo being tested, and may consequently turn to medical care only in advanced stages, resulting in poorer outcomes. This may potentially also put their families and communities at risk.

A recent commentary about the COVID-19 response in Iran [3] exemplifies the concerning general response capacity of low and middle income countries (LMIC) [4], and the barriers faced concerning testing, provision of protective personal equipment (PPE) for health staff, and advanced medical services. People living in LMICs, as well as millions of poor people living in high income countries, also experience significant barriers to self-protection due to crowdedness, poor housing conditions and poor sanitation. Additionally, the global economic downturn and limited international support puts millions of people in LMICs at significant risk to being food insecure. There is also a lack of adequate control of additional outbreaks. These actual and potential perils have pushed the World Health Organization (WHO) to increase support to these countries to improve resilience and reduce the health and social impact of future health emergencies [5].

It is of increasing concern that the current COVID-19 death toll fails to capture the devastating collateral effects on health equity. This includes the loss of health insurance, jobs and homes, which increases risk for mental and physical morbidity and all-cause mortality. Social isolation and movement restrictions also jeopardize health and access to basic needs, and point to the need for human rights protection and the development of public health and social measures that are rolled out in a comprehensive manner and without discrimination against vulnerable populations, including refugees, undocumented immigrants and people in detainment and correctional facilities [6, 7]. Thus, comprehensive responses that take into consideration the complexities involved in protecting human rights, including the protection of humanitarian workers [8] need to be systematically implemented.

To address the effects of the current pandemic on global health equity we have compiled 13 country case studies highlighting the unique needs of vulnerable populations and some of the measures taken to address these needs by each country. As information on the global public health response and consequences of COVID-19 is yet to be systematically collected and reported, the case studies presented here provide an interim account of challenges and some emerging solutions, which can potentially be more widely disseminated as countries realize the need to address COVID-19 related health equity issues even after containment of the outbreak. Our team of authors represent expert research and public health professionals in the topics they cover. For a more comprehensive account of publicly reported country data (not specifically focused on health equity) readers are encouraged to turn to the WHO situation and country reports, and to resources such as cross country analysis provided by the Health System Response Monitoring initiative (https://analysis.covid19healthsystem.org/).

Additional commentaries and research letters, recently published at the IJEqH, add to the growing body of knowledge on COVID-19 related health equity issues and local, country and regional responses. In a commentary from Italy, authors address the perils of discrimination against the elderly in regards to ICU admission criteria and social distancing measures [9]. A study from Mexico showcases a methodology which may be used by other countries proposing interventions to protect vulnerable families with young children from the negative socio-economic impacts of social distancing measures required to tackle COVID-19 [10]. Spatial inequality is addressed in a recent letter from China on how “pairing assistance” was used in order to mobilize personal to areas hard hit by COVID-19 [11]. A commentary on the challenges facing indigenous communities in Latin America draws a practical guide for designing and implementing a response to COVID-19 in indigenous communities [12]. Lastly, a commentary on mobile health clinics in the US shows how they can be used to overcome barriers to access that are experienced by under-resourced communities in general, and at times of crisis, such as the COVID-19 presents, in particular [13]. We encourage authors to continue submitting health equity research papers and commentaries in order to increase global knowledge and allow cross-national learning of what works for those most at need due to the direct and collateral effects of COVID-19 on health equity worldwide.

### COVID-19 health equity perspectives: China

#### Yingyao Chen

COVID-19 pandemic and its successful control and management in China demonstrates that health is at the top priority in response to the serious disease outbreak consequences, compared with economic implications, such as GDP growth rate. In the path of universal health coverage in China, almost all people are covered by the employee urban medical insurance scheme, the urban and rural resident medical insurance scheme or the rural cooperative medical scheme, however, participants of those schemes have different benefit coverages reflecting equity challenges. According to Andersen’ Behavioral Model of Health Services Use [14], enabling resources is one of important factors to influence equity, so health insurance and its intensive and extensive coverage is key for vulnerable population to access to care.

Dr. Tedros Adhanom Ghebreyesus, director-general of WHO, stated that “equity is critical. This applies to information, innovation, essential medical equipment and supplies, as well as medicines, vaccines, and diagnostics [15].” In the epidemic of COVID-19, financing for health services is also very important to equitable to access, especially in developing countries.

For example, in Wuhan, patients who suffered from COVID-19, no matter if from urban or rural residence in Wuhan, hometown in Wuhan or beyond, were covered by any of the medical insurance funds and government subsidies, which averaged 65% insurance rates. Due to services provided to people with COVID-19, hospitals have exclusive insurance funds beyond their usual budgets so that they don’t have any financial concerns to accept patients or serious cases in need of ICU. Regarding pharmaceuticals and services for treatment of COVID-19 which were not included in the national drug/services reimbursement lists, they were temporally granted reimbursement [16]. Special equipment and services, such as -Extracorporeal Membrane Oxygenation Machines (ECMO), equipment for emergency services for very serious COVID-19 patients, were included in reimbursement schemes as needed. In addition, any medical insurance payment included also complicated procedures, and bureaucracy has improved much in the period of COVID-19, so individual patients don’t need pay the bill and medical insurance authorities in both regions settle down the transactional payment. Such processes reflect that streamlining of payment in different regions is feasible and implementable. Medical insurance reimbursement policies significantly improved access to COVID-19 treatment in China, and it sheds light on the future development of universal health coverage in the context of health care reform in China.

In addition, caring for disabled people is quite challenging, and Shanghai implemented many measures to meet their needs in the period of COVID-19. The civil services authority classified the disabled, contracted with 3rd party companies, and arranged home nursing care for those high-need people. The local community health centers provided health services for the disable people, including home-visits, online-visits, prescribing, delivery of re-filled drugs, and others. The local citizen organizations with volunteers facilitated other social services if requested, such as food and daily necessities delivery services. It is quite important for those disabled people to access health and social services, and local community organization, the disabled person union, NGO and volunteers played key roles in addressing these needs.

### COVID-19 health equity perspectives: Brazil

Luis Eugenio de Souza, Inês Dourado

COVID-19 outbreak officially reached Brazil on February 26th, and the first COVID-19-related death was notified on March 17th in São Paulo state. On March 20th, the Brazilian Ministry of Health (MoH) declared the existence of community transmission in the entire territory. On May 13th, the country had accumulated 177,589 confirmed cases of COVID-19 (84.5 cases/100.000 inhabitants) and 12,400 COVID-19-related deaths - fatality rate of 7.0%. A week later, almost 60.000 cases were estimated, One of the highest transmission rates of the virus in the world [17].

The coronavirus arrived in Brazil by plane, brought by people from the upper classes from Europe, mostly white and highly educated. Once landed, it contaminated domestic workers, generally black and less educated people who, then, disseminated the infection to their poor communities. Given this pattern, the rates of infection and case fatality recorded in China and Italy may not be adequate parameters to anticipate the trajectory of COVID-19 in Brazil, the 9th most unequal country in the world [18].

On March 11th, the MoH published guidelines on the isolation of those infected with or under clinical or laboratory investigation for COVID-19. Recommendations of quarantine were first made by state governments with worst scenarios, and later became a MoH recommendation for the whole country. Although the MoH has also recommended quarantine measures, the country’s President, Jair Bolsonaro, has insisted on a denialist approach, making public announcements contrary to the scientific evidence existing so far. He has publicly insisted that there is not a public health emergency in place, and has encouraged people to not respect quarantine orientations, alleging that it is necessary to preserve the economy.

The COVID-19 pandemic placed health services under considerable strain and this situation can be worsened in developing countries. Brazil has a public, universal health system known as Sistema Único de Saúde (SUS) established as a universal right of citizens, and a responsibility of the state, under the Federal Constitution, enacted in 1988. SUS is at the forefront of the response to the pandemic. The system, however, has been historically underfunded and particularly harmed by austerity measures implemented in 2016, when public investments in social areas were frozen for 20 years [19].

In general, Brazil has a high number of ICU beds (2.03 per 10,000 inhabitants), even comparable to that of developed countries. The distribution of beds between the public and private systems, however, reveals, social inequalities. While, SUS has 1.3 ICU beds for 10 thousand inhabitants, the private sector offers 4.45 for 10 thousand beneficiaries [20]. In view of this situation, the Brazilian government, like those of other countries, would be expected to mobilize or request private services to act in a collaborative and coordinated manner with public services. However, the opposite is true: the federal government has not required any private sector participation. Furthermore, inequities are observed at the regional level. The Southeast region, the richest in the country, has 2.58 ICU beds per 10,000 inhabitants, while the poorer North and Northeast regions have 1.23 and 1.35 ICU beds per 10,000 inhabitants, respectively.

Considering that 30 million Brazilians do not have basic sanitation [21], it is easy to understand that the simplest recommendations - such as washing hands frequently or using alcohol gel - are difficult to be carried out for a large proportion of the population. Likewise, it is almost impossible to avoid crowding or to recommend home-office working for the 11.4 million people who live in slums [21], sharing cramped and poorly ventilated houses and with needs to go out to manage their daily lives. Therefore, the risk of COVID-19 may be greater among the poorest Brazilians reflecting socio and economic inequalities.

Furthermore, this economically disadvantaged population is the one with the highest prevalence of chronic conditions such as diabetes, hypertension, cardiovascular and respiratory diseases. In fact, the National Health Survey (2013) revealed that the proportion of people with one or more of these conditions represents 54% from the group that only attended elementary school versus 28% from the group that attended high school and 34% from the best-educated group [22]. And it is known that the presence of comorbidities is a strong predictor of COVID-19 severity.

Racial inequality is also an important issue in Brazil as the majority of poor people are black. Moreover, it is already playing a role during the pandemic as the higher case fatality rate by COVID-19 is among blacks. Although Blacks represent 23.1% of hospitalized Brazilians with COVID-19, 32% of them died, and among whites, the situation is reversed, as the proportion of hospitalized cases is 73.9 and 64.5% died [23].

Indigenous people are also a group of those most vulnerable to the COVID-19 pandemic due to worse social, economic and health conditions than non-Indigenous, which can increase the risk of the disease. In addition, barriers to access health services due to geographic distance or the unavailability of health services represents yet another disadvantage of this population.

Two other sub-groups of the population in a situation of vulnerability are noteworthy: prisoners and homeless people. Brazil has the third-largest prison population in the world, 773,000 prisoners. It is estimated that 80% of the prison population will be infected with the virus, of which 20% may need hospitalization and 10,000 (8%) may need an ICU bed [24]. So far, initiatives taken to protect this population have been limited to suspending visits and temporary exits, and general guidelines on health care issued by the MoH. Not surprisingly, several rebellions are taking place in prisons. According to the Institute for Applied Economic Research (Ipea), in 2015, there were 101,854 homeless people in Brazil [25]. They are invisible to social policies, even to emergency measures as the small allowance of 100 US$ a month, for 3 months, which the National Congress approved and the government is being slow to pay.

Finally yet importantly, Brazil may face difficulties in responding to other health problems, such as those related to sexual health during the pandemic. Experience from past outbreaks indicates the need to pay attention to the potential effects of the COVID-19 pandemic on sexual health outcomes, both at immediate and long terms [26]. Moreover, little is known about the effects of COVID-19 on sexual health and prevention of HIV and other sexually transmitted infections (STI) so far, and the limited available data requires cautioning against early assumptions. Potential negative impacts of COVID-19 that may be anticipated include a shortage of provisions, such as condoms, antiretroviral drugs for HIV/AIDS and antibiotics to treat STI; diversion of healthcare providers to help address the epidemic; and diversion of financial resources to COVID-19 response, reducing the availability of sexual healthcare delivery. In Brazil, the provision of minimum healthcare services in all areas during the pandemic has been recommended, with federal recommendations on maintaining the delivery of HIV testing, STIs diagnosis and treatment, and regular supply of HIV pre-exposure prophylactics (PrEP). However, even when services are available people may be unable to go to healthcare facilities during the pandemic because quarantine can lead to economic hurdles; or they may avoid doing so as means to avoid crowds10 or to avoid disclosing their sexual health needs to people within their household. Yet, quarantine may result in changes in sexual behavior, which can consequently affect the use of preventive methods.

In sum, the social, racial and regional inequalities present in Brazil are revealing themselves in rates of infection and lethality, as well as in the possibilities of conforming to quarantine and other preventive measures, and in access to intensive care health services.

### COVID-19 health equity perspectives: Thailand

#### Piya Hanvoravongchai, Rapeepong Suphanchaimat

With the ongoing global challenges posed by COVID-19, Thailand health systems seem to be in good control of the situation considering disease containment and medical care responses. As of 18th May, the Ministry of Public Health reported 3028 confirmed cases or less than 50 cases per million population, and 56 deaths [27]. Initial responses in the medical care system may seem somewhat chaotic. Inadequate surgical masks and personal protective equipment for frontline health care workers, were met with public outcry and it incited strong citizenry-based responses through various donations to support the health workers. Over time, better coordination and communications eased the tensions and strengthened Government’s response to help improve the epidemic situation.

Thailand’s relatively strong health care delivery system and its near universal coverage of health insurance in the country have contributed to such achievement [28]. It is considered among the top 10 of the most prepared countries and ranks the first among all upper-middle income countries based on the 2019 Global Health Security Index [29]. The extensive network of health care facilities and primary health care, majority in the public sector, contributed to the success of case finding, contact tracing, and containment efforts. Local health officers and village health volunteers worked in consort with the Ministry of Public Health’s investigation team in performing active case finding and contact tracing, and were active in promoting public health measures and messages for social/physical distancing, including providing support to those traveling back to their local community, after the partial closedown in big cities, to complete their quarantine requirements. Existing near universal health financing coverage allows the Government to quickly offer free COVID-19 tests and medical care, through the three major public health insurance schemes in the country, resulting in no financial barriers to necessary tests and health care access.

The COVID-19 pandemic is in its initial months and it is still a long way to go. Nevertheless, the experience in Thailand already revealed several areas for improvement of our health care systems. One vulnerable population is the overseas migrant workers which Thailand has over four million of them [30], many entered the country illegally and do not have health insurance coverage. Their working and living conditions are conducive to exposure and spread of the infectious disease. Though the Ministry of Public Health sent a message to the wider public that all migrants regardless of their legal status are allowed to enjoy COVID-19 test and treatment without financial barriers, such policy message is not effectively operationalized due to various reasons, including varying interpretations on the rights of migrants, let alone the bureaucratic constraints. To strengthen pandemic responses and mitigation, better coordination and sharing across health information systems will be necessary.

The bigger challenge to health equity will likely stem from the social and economic implications from the pandemic and its policy responses such as partial lockdown restrictions. Potential delay in treatment may result in an increase in complications and severity of diseases, particularly for chronic disease sufferers of the poor and marginalized populations. Inadequate social protection coupled with inadequate social support from the government to mitigate the impacts of economic recession and rising unemployment means more households and children will be pushed into poverty. From the World Social Protection Report 2017–2019, the level of allowance for old age pension was very low and inadequate despite its universal coverage, and nearly four million children were still not covered by any social protection such as child support grant [31]. A rapid assessment of non-Thai populations identified job loss and inadequate income to cover daily living expenses as their main challenges [32]. Lesson from previous economic crisis shows that physical and mental health problems can potentially rise, especially among the poor and vulnerable populations [33].

The news media started reporting on long queues of people lining up for food and necessity donations in various communities in the country. It is a demonstration of social solidarity but charity alone will not be able to solve the upcoming social and health problems especially during the challenging times. Thailand has made good systematic progress towards universal health coverage. The current crisis can serve as an important opportunity to mobilize public support to narrow the gaps and strengthen national policies to address social determinants of health and universal social protection, an imperative for better equity in health and social justice.

### COVID-19 health equity perspectives: sub Saharan Africa

#### Peter Hangoma

The burden of deaths from covid-19 has been unequally borne by old people and those with pre-existing chronic conditions [34]. Faced with just over 16,200 cases and 873 deaths as of April 16th, which accounted for less than 1% of global infections and 0.6% of deaths, the corona virus cases in Africa have grown faster than the global average, increasing almost 5 folds in Africa, standing at 75,380 as of May 16 -- many times higher than the global average growth. Deaths have increased 3 folds with 2563 confirmed deaths by May 16. While it is noteworthy that drastic interventions have been taken by governments in Africa, like in other parts of the world to contain the virus [35], governments are beginning to relax these interventions given the discussion that such interventions may cause more deaths from hunger and other diseases than the Corona virus itself. A careful application of interventions and lockdowns is still required to slow down the rate of transmission to levels which health systems can handle, while at the same time avoiding excessive lockdowns that may hurt the poorest. Although discussions are beginning to emerge, there has been less attention on how covid-19 and the accompanying interventions may disproportionately affect the poor and other vulnerable groups [36], and what measures can be put in place to mitigate their suffering. A focus on equity is important given that reducing inequality is one of the fundamental sustainable development goals (SDGs). Unlike the high-income countries of Europe, North America, and Northern Asia, most countries in sub-Saharan Africa (SSA) already bear a substantial burden of infectious diseases like HIV/AID, Malaria, and Tuberculosis [35]. The burden of chronic conditions is also rising. Worse still, over half of its population live in poverty with poor living conditions and limited access to social services and amenities. Most countries have weak health systems. The coronavirus and measures taken to contain it may have serious equity effects.

First, poor households, who constitute the largest proportion of the population in SSA, are likely to be hit the hardest by covid-19 because the celebrated interventions of social distancing and staying at home are unlikely to be effective [35]. This is because the majority live in crowded conditions implying that the infection rate, or the average number of people that each infected case will infect, in such crowded conditions is likely to far above the estimated 2–4. Serious efforts are needed to interrupt the spread to these communities.

Second, supply and demand for other health services is also likely to be affected [37], disproportionately affecting the poor. On the supply side, with so much effort concentrated on the fight against covid-19, there are concerns that other health services may suffer. With limited resources, most countries in SSA will divert funds to the fight against covid-19 and neglect other conditions such as malaria, maternal and child health conditions, and tuberculosis which account for some of the highest death rates among the poor and account for a substantial share of the disease burden. For example, In Zambia, like many African countries, maternal and newborn conditions, cause substantial mortality, are costly [38], and access to health facilities is important. Previous studies have shown that during pandemics, all-cause mortality rises substantially [39]. Since the burden of these conditions is already unequally borne by the poor, the covid-19 pandemic is likely to reinforce these inequalities. On the demand side, people are likely to shun or forego care. This is likely due to the fear of contracting coronavirus in health facility or a result of perceived reduction in the quality of health services as health resources are concentrated towards the fight against covid-19.

Third, although primary health services are said to be free at the point of use in Zambia, like many other SSA countries, there are other costs that must be borne by patients who want to access health services. These include transport cost as well as costs of medicines and diagnostics that are not provided in health facilities. These costs are likely to rise as covid-19 spreads and poor households in urban slums and rural areas will likely constitute a disproportionately higher share of the financial risk as they attempt to seek treatment or self-medicate. This will raise the risk of catastrophic spending and impoverishment, driving them deeper into poverty.

Fourth, and last, inequalities in nutrition as well as access to food and other services are likely to be reinforced by lockdowns which restrict business activity of the majority of the population [40]. More than 85% of the population in SSA are employed in the informal sector, mostly depending on daily income for survival of their families and have limited or no access to social protection. In addition, the nature of these jobs is such that they cannot be done from home. In Zambia, the closure of mines, restaurants, bars, and other supporting businesses has sent a lot of people home without any benefits, as is expected in informal employment. In addition, there is anecdotal accounts that the general economic environment cause by lockdowns has forced small scale traders and marketeers, transporters, etc., to close their business which has subjected their families in economic hardships.

### COVID-19 health equity perspectives: Nicaragua

#### Claudia Obando

While the rest of the world is receiving warnings about the coronavirus, the Nicaraguan government has not been imposing measures to prevent spread of the virus. Considered the second poorest country in Latin America, the health system is a big problem not only because of the conditions of the public hospitals, but also because of the catastrophic of the public health system response. Knowing that prevention could help mitigate impact of the pandemic, the government ignored this, risking the health of people by promoting Easter celebrations and gatherings on beaches.

According to official figures Nicaragua has 25 confirmed cases of the COVID-19 (May 19th), but the government reports have been criticized from different institutions, including PAHO and Human rights organizations. Criticism stems from the acknowledgement of how rapidly COVID-19 can spread, especially if people are not taking precautions, and from comparison to neighboring countries, which closed the borders since the first cases were detected in Central America.

Besides the negligent actions of the government and the poverty, the public have been taking their own safety measures, by fabricating facemasks, promoting hand washing, staying at home and not sending children to school -- all individual decisions based on recommendations spreading especially from social media.

### COVID-19 health equity perspectives: Armenia

#### Varduhi Petrosyan

Armenia is a small country with a population of three million; it allocates around 1.5% of its GDP as tax-based public expenditure on health. However, public health efforts, primary health care, emergency care, and care for infectious diseases is currently fully covered by the government from general taxes.

The Armenian government started working on this issue in January creating a special government intersectoral commission to develop and implement strategies to address the situation with SARS-CoV2. On March 16, the Armenian Government declared a state of emergency, a week or so later a complete lockdown. A major public awareness raising campaign started about the virus, how to prevent the spread of the virus, about what is lockdown, self-isolation, quarantine, etc. The majority of citizens trust the current government in Armenia and it helps to manage the pandemic situation much better. Government is very transparent about the situation in the country. Different branches of the government continuously have press conferences to inform the public about the current situation and 18 different programs that the government developed to help socially vulnerable households, daily wage earners, those who lost their job because of the pandemic, and small and medium size businesses, particularly those who did not lay down their employees. The Committee and all government agencies have widely shared their hot line numbers to be accessible to the citizens.

Moreover, the Government, in collaboration with the IT sector, developed an Application on COVID 19 symptoms for self-diagnosing and giving advice to those who seem to be at risk on how to contact primary healthcare providers, and sending an alert to the National Center for Disease Control and Prevention about a need to do testing. It also provides latest official news and advice on infection control. The Government is also using mobile phone tracking to strengthen the contact tracing efforts.

Government organizes charter flights to ship newly purchased medical equipment, medical masks, PPE, PCR tests, and other necessary materials. Government is also supporting local production of medical masks and diagnostic tests to improve access to those goods.

The Government, including the Ministry of Social Protection, has changed the way they distribute pension to make sure that the pensioners do not leave their house to get their monthly pension. Pension is being taken to their apartments/houses.

The Ministry of Health (MOH) and the Government came up with different plans for different stages of the epidemic in Armenia. The MOH first changed the Infectious Disease Hospital to one accepting only COVID 19 patients. In parallel, nine other hospitals/clinic – five in Yerevan, the capital city, and four in the other regions of Armenia – were renovated, furnished, and equipped to accept only COVID 19 patients. One of the biggest state-owned multidisciplinary hospitals was also reprofiled for COVID 19 patients only providing an opportunity to have appropriate care for patients with multimorbidity. In addition to this, to be ready for a worst-case scenario, in Yerevan and the regions several big sports/concert halls are prepared to admit patients if the number of patients goes up disproportionately. The MOH has been expanding the lab capacity in the country to be able to do more testing per day without compromising safety of the staff.

Algorithms have been prepared for the Primary Care Providers to be able to respond to the situation properly, making sure everyone has good access, but without putting others into danger. All the primary care facilities have been working extended hours with 24-h functioning hot-lines. The phone numbers of all facilities have been widely circulated among the citizens, so that they could contact primary care providers and check with them about appropriate steps. Primary care providers in consultation with the National Center for Disease Control and Prevention make decisions when a special ambulance service should transfer the patient to a specialized facility for diagnostics and care.

Moreover, hot line numbers of the Ministry of Health and its National Center for Disease Control and Prevention have been widely circulated via TV and social media to make sure all citizens have equal access to the existing resources.

So far, all the confirmed cases, even without symptoms or very mild symptoms have been put into either hospitals or special hotels to fully isolate them and prevent the spread of the virus. Armenia has a supply of all those medications that many countries have tried for treating particularly severe COVID 19 patients.

The National Institute of Health of the Ministry of Health has been conducting large scale trainings of physicians, nurses, and lab technicians. In collaboration with the Diaspora High Commissioner’s Office continuous webinars are organized between Armenian and diaspora health professionals to share their experience with each other.

Starting March 1, when the first case was identified, the Ministry of Health started rigorous contact tracing and quarantining all exposed persons, and later all travelers coming to Armenia from different countries. The Government of Armenia mobilized many hotels for these people under quarantine. The conditions, including food, and other amenities have been very good and safe equally for everyone. The staff organizing quarantine have done a lot to protect the dignity and mental health of people. Later, the Armenian Government organized charter flights to bring its citizens from neighboring countries, most of them were migratory workers who were left without housing and jobs because of the pandemic. Many of these citizens are now in Armenia, under 14 day quarantine that is fully supported by the government, at 0 cost to the citizens, in safe and good conditions.

Social solidarity: during the pandemic the regular citizens made donations to a special account to get additional medical resources needed such as PPE, equipment, tests, and for making extra payment to the frontline health providers. Thousands of citizens and diaspora contributed financial resources for this purpose.

Thousands of volunteers got together to offer help and support to older age citizens of Armenia who are at higher risk for COVID 19. They prepared packages with food and other necessary items for 1 month or 3 months, so that our older age people do not go to stores. The priority has been given to those older age people, who live alone.

The international agencies have also mobilized resources to help Armenia. Several countries made donations of medical tests and other needed supplies.

Concluding remark: In my judgement, the Armenian population did not face inequity in access to information, to safe and appropriate quarantine services, access to diagnostic tests and care for COVID 19. Given the available scarce resources, I think, the Armenian Ministry of Health and Government have done a tremendous amount of work with great commitment to protecting wellbeing of their citizens.

My concerns are related to health consequences of economic downturn and widening income inequality in societies. Moreover, with major global disruptions in flights and transportation of goods, there might be disruptions of medical supplies, and those with multiple chronic conditions, one of the most vulnerable groups, could suffer the most because of these disruptions. The inequity between high-income vs. low- and middle-income countries will become even more concerning. I think, all the countries should collaborate and unite their efforts to mitigate the consequences of this pandemic.

This pandemic demonstrates very vividly the need for universal health coverage, the benefits of having strong public health services, and the importance of social solidarity.

More information about Armenia’s response is available at: COVID 19 Health System Response Monitor: Armenia https://www.covid19healthsystem.org/countries/armenia/countrypage.aspx?fbclid=IwAR01MvAuqMrRF2VOfW_mrFlW_7tzgE6a_2Km71X-Jb0I33aCLAQxoLY481M

### COVID-19 health equity perspectives: India

#### Krishna D. Rao

India has experienced relatively lower levels of morbidity and mortality due to the COVID-19 pandemic, though both confirmed cases and deaths continue to increase. The population shutdown to control the Coronavirus disease (COVID-19) pandemic has had devastating effects on the livelihoods of economically vulnerable populations. In India, just after a national lockdown was announced on March 22nd, thousands of migrant workers in cities found themselves without an income source and unable to sustain themselves started returning to their villages. In general, unemployment has increased substantially, particularly among those in the informal sector. It is now increasingly clear that economically vulnerable individuals are being forced to sacrifice disproportionately more for public health measures that benefit all of society. Further, the lockdown has also made it difficult for sick individuals to access health services.

Population lockdown and the associated loss of economic activity has affected employment and livelihoods on a massive scale globally. India has the largest number of people under lockdown anywhere. By all accounts this lockdown has had a significant effect on movement of people, job losses and incomes. According to information provided by the Center for Monitoring the Indian Economy (CMIE), in mid-May the unemployment rate was 24, and 44% of households currently reporting a loss in income, up from around 10% in early-March. A recent telephonic survey carried out by National Council for Applied Economic Research (NCAER) in Delhi found that 84% of respondents reported loss of income, and nearly 30% experienced shortages of food, fuel, and medicines; these outcomes were concentrated among the poorer households and in rural areas. Historically, economic downturns are associated with the loss of livelihoods, decreases in dietary intake, poorer dietary quality, and health care consumption. The closing of schools due to the lockdown has deprived many children of their only nutritious meal through school-feeding programs. Moreover, children who experienced poor nutrition in utero or during their early years have long term effects on future cognition, educational achievement, and adult income.

The Indian government, in recognition of the hardship imposed on economically vulnerable groups, has announced several relief measures. In mid-May the government announced a $260 billion rescue package for the economy, though its details are not clear. As part of this, the government said that it will provide free food grains to migrant workers for 2 months, as well as, subsidized loans to those in the informal sector and small farmers. Earlier, the government announced direct cash transfers (a one-time cash transfer of $13.31 to 30 million senior citizens and $6.65 a month to about 200 million poor women for next 3 months), as well as, make free food (wheat, rice, pulses) available to them for the next 3 months.

While the relief measures announced by the government are important and significant, it’s important to be cautious. The effectiveness of these safety nets will depend on the adequacy of the relief package, how well they reach the neediest groups, and efficiencies in delivery system. It is well known that safety net programs in India are generally inefficient and often miss the poorest. Secondly, it is important to note that these are one-off relief measures intended only for a short period of time. The economy will take some time to recover and the associated deprivation will linger long after the pandemic is over. Critically, the economic and health deprivation caused by COVID-19 will have long term effects. For example, pregnant women and young children who have experienced illness and/or poor nutrition in this period will likely suffer long term effects its terms of reduced cognition, educational achievement, and adult income.

Epidemics have been particularly harsh on the economically vulnerable. One of the most powerful articulations of this was by Rudolf Carl Virchow, a prominent German biomedical scientist of the nineteenth century, who writing about Typhus outbreak in Upper Silesia (in Poland) remarked that the “population had no idea that the mental and material impoverishment to which it had been allowed to sink, were largely the cause of its hunger and disease, and that the adverse climatic conditions which contributed to … the sickness of its bodies, would not have caused such terrible ravages, if it had been free, educated and well-to-do.” Governments need to prioritize the economic security of those affected by corona virus control policies, as much as, ensuring that health systems are adequately resourced to manage the COVID-19 outbreak.

### COVID-19 health equity perspectives: Guatemala

#### Ana Lorena Ruano

The Guatemalan government has provided a weak and incoherent response to the threat of COVID-19 leaving, once again, the most vulnerable and marginalized populations even more unprotected. Although several weeks have passed since the first case of COVID-19 was identified, the government and public health authorities refuse to release detailed information about the numbers of cases, number of tests carried out, and their distribution. They are also unclear about the links between identified community cases, with negligible levels of contact tracing. Additionally, the Guatemalan army has been deployed to create barriers around communities that have a community case identified, and do not allow food or any resources inside. This will inevitably lead to the loss of human life among the most vulnerable and poor populations.

Incoherent policies aimed at protecting the interest of the business interests of the Guatemalan chamber of commerce (CACIF) have caused much confusion over curfews, medicine, correct reporting behavior, and most importantly, how to treat those identified as testing positive for COVID-19. Neighbors stormed the house of a women and her five-year-old child in Escuintla, with the intent to lynch them for bringing the disease into their community [41].

Weak health system responses have only increased the precarious situation of Guatemalans. With less than 400 intensive care beds, and fewer 50 respirators, the country is woefully unprepared for the wave of cases that will arise in the following weeks. Two reference hospitals had already collapsed in the first half of May, long before the pandemic hit its peak in the country. As a response, the president and physician Dr. Alejandro Giammattei, has alternatively referred to the virus as a simple flu, as something that would disappear as the country’s temperatures rose, and later as a matter of personal responsibility. It is unreal to believe that a Guatemalan citizen can maintain social distancing when the government forces essential businesses to open during very restricted hours, does not provide the social and economic support that is needed, and changes the policies and rules around the curfews almost daily.

Despite strict rules, life in Guatemala City remains pretty much the same: long hours spent in traffic and crowded streets, with authorities being lenient with the curfew on pay days and reports of drunken congressmen and police officers disregarding social distancing orders.

The presidential decree that closed down public transport left hundreds of thousands of Guatemalans that live in rural areas and small hamlets without communication to the outside world. With no trucks or busses, food has quickly become scarce and prices have hiked. In addition, the closing of the schools also limits the ability of many families to secure even one meal a day for themselves or the children. This is a country were 54% of the U5 children are chronically malnourished, and where almost 80% of indigenous people are poor. As of May 15, 2020, malnutrition had tripled among U-5 children.

Guatemala agreed to become a “third safe country” for the United States, and the country is expected to host the asylum seekers that are fleeing the same conditions of violence, structural poverty, and exclusion that caused them to flee in the first place. In addition, the US has not stopped deportations and outside reports indicate that about 75% of deportees test positive for COVID-19 [42]. Many have not been allowed to return to their communities and when they do, they are run out. This puts many families at increasingly and alarmingly high rates of vulnerability as they stop receiving remittances from abroad and are unable to have this breadwinner working in their land.

The opposition has demanded explanations from the government and the Ministry of Health. During a hearing held before the identification of the first COVID-19 case in the country, MoH authorities were asked to present their strategy. In response, a vice-minister of health reported that there was no formal plan to deal with the COVID-19 threat because “Guatemala is a land loved by God”. When an openly gay congressman demanded explanations from the president, the non-practicing physician, Dr. Giammattei responded using vulgar and homophobic remarks, and declined to give more information. Since then, the MoH has reported that numbers are low because “God’s finger is on Guatemala”.

A multimillion-dollar relief package was intended to help small businesses, individual families and people in the informal economy, which represents about 70% of the Guatemalan workforce. However, after the CACIF lobby, these measures where changed to benefit large businesses and banks, and the president vetoed the bill meant to provide relief from eviction and from basic services like water, internet and electricity being cut off. Municipal governments were asked to identify which informal workers are worthy of the government subsidy, and reports from human rights defenders show what was expected: these lists of workers are highly politized and only carried out in areas where the president has support from municipal authorities. This has great implications for local grassroots associations, many of which face considerable pushback from their local governments. The subsidy will be used as a political tool to benefit supporters and punish dissenters. In addition, only people with electricity in their homes will receive the family help.

Guatemala, like the rest of the northern triangle in Central America, is set to become a killing field. With no health system resources, weak and incoherent government policies, and authorities interested in serving the economic elite over the poorest and most vulnerable of Guatemalans, there is little hope that the death toll will not be in the thousands.

### COVID-19 health equity perspectives: USA

#### Leiyu Shi

The high volume of coronavirus cases the U.S. is now facing, coupled with shortages in medical equipment and supplies, demonstrate the chilling consequences of neglecting public health and preparedness in fighting against pandemics such as COVID-19 and the disconnect between public health and healthcare delivery. Lack of preparation and lackadaisical attitude towards the outbreak led to an inability to take action early on. The U.S. is now the country with the most COVID-19 cases and deaths in the world, despite having weeks and even months to prepare.

Ensuring the safety and well-being of vulnerable populations during the COVID-19 pandemic has presented a big challenge. These include the impoverished, the disabled, refugees, minorities, the uninsured, and other marginalized communities who have historically paid the highest prices in terms of health and welfare during infectious disease epidemics. They face significant challenges in gaining access to equitable care and staying economically and socially stable during a pandemic. Because of their higher rates of chronic disease, they are also at higher risk of developing severe symptoms if they contract the virus. Many vulnerable individuals do not receive paid sick leave or any type of paid leave that would make caring for a sick family member or a child home from school economically viable. Others may have lost their jobs as a consequence of the pandemic, making it more difficult to access quality, nutritional food, and safe, affordable housing [43].

COVID-19 cases have skyrocketed among vulnerable populations. In Chicago, for instance, African Americans account for over 50% of all COVID-19 cases. The infection rate in predominantly black counties in the U.S. is also over three times that of predominantly white counties [2]. The Native American population is also at severe risk – the Navajo Nation currently has more per capita cases of COVID-19 than any state in the country. With high rates of chronic disease, shortages of medical personnel, and poor access to basic necessities such as running water, Doctors Without Borders has dispatched a team to help mitigate the unfolding Navajo health crisis. This international humanitarian organization, which typically works in war-torn regions around the world, has never sent teams to the U.S. prior to COVID-19 [44].

Additionally, America’s homeless population faces major problems with regard to access to health care and an elevated risk of community transmission [45]. The uninsured also face significant barriers in accessing care since they have no established provider-patient relationship or health care network. America’s 2.3 million prison population further presents a challenge. Correctional facilities do not have the medical capabilities to treat those who fall seriously ill from COVID-19; prison health care systems are also generally understaffed and ill-equipped to handle a surge in patients [46]. A high prevalence of chronic disease and elevated risk of transmission from being in such close, enclosed quarters make these facilities high-risk breeding grounds for virus transmission [47].

In terms of testing and treatment costs, major insurance companies have waived copays for those insured [48]. For the uninsured, the federal government has agreed to pay doctors and hospitals at Medicare reimbursement rates for treating COVID-19 patients provided that they accept the payment as full (in order words, they cannot ask patients to pay for the difference) [49]. For businesses, and in particular small business and those unemployed due to COVID-19, the U.S. Congress has so far passed four pieces of legislation. The Coronavirus Preparedness and Response Supplemental Appropriations Act provides $8.3 billion in emergency funding to federal agencies [50]. The Families First Coronavirus Response Act provides paid sick leave, tax credits, free COVID-19 testing and expansions to food assistance, unemployment benefits, and Medicaid funding [51]. The third bill, the Coronavirus Aid, Relief, and Economic Security Act (CARES), is a $2.2 trillion stimulus package that includes loan programs for businesses, increased unemployment insurance, health care funding, direct payments to adults under a certain income threshold, and state/local aid [52]. An additional $484 billion relief package for small businesses and hospitals was passed at the end of April [53]. Further legislative action is continuing.

To bridge the gap between public health and health care delivery, the federal government set up the COVID-19 Task Force headed by the Vice President. Along with FEMA (Federal Emergency Management Agency) the Task Force is in charge of leading the nation’s public health response and securing resources to distribute to states [54]. FEMA is also helping lead the Army Corps of Engineers in setting up emergency medical infrastructure and the Department of Transportation in supporting supply chains [55].

To enhance production of Personal Protective Equipment (PPE), the Trump administration has invoked the Defense Production Act (DPA), which allows the federal government to order the domestic production of necessary equipment in times of crises. The DPA has so far been used to command domestic companies to produce crucial equipment including ventilators, masks, and testing swabs. The Department of Defense has started handing out federal funding contracts to boost such production [56]. To address local shortage in health system capacity, the federal government has enlisted the U.S. military to assist state and local governments. The military’s two hospital ships have been dispatched to New York and California, respectively, to provide extra beds and facilities; all states have also activated the National Guard. The Army Corps of Engineers has been dispatched to multiple states to construct temporary COVID-19 hospitals. Army field hospitals are also being deployed across the nation to provide additional care [52].

### COVID-19 health equity perspectives: Israel

#### Efrat Shadmi, Sivan Spitzer-Shohat, Inbal Faran-Perach

Israel has universal health coverage through its National Health Insurance (NHI) law with four health funds operating as insurers and providers of services and a mix of government, public, private and health-fund owned and operated hospitals. A relatively quick governmental response to the COVID-19 threat, in terms of border closures and stay-at-home recommendations is considered to have helped Israel achieve relatively low confirmed cases and death rates to date (1901 cases/1 m population and 29 deaths/1 m population as of May 10th). Early on, the Israeli healthcare response has mainly relied on Magen David Adom (MDA, Israel’s national, ambulance and blood bank service) for performing testing, general hospitals which have almost all opened or built new designated COVID-19 units, and the four health plans which have all launched a primary care response for remote monitoring of low acuity patients. Health plans are charged with overseeing care to COVID-19 patients who are confined to their home or are transported (by the MDA) to designated hotels, currently constituting about 2/3 of all known cases. Such a widespread primary care response is called for especially given the limited hospitalization capacity, with a severe shortage of hospital beds (2.2/1000 compared to 3.6/1000 OECD average).

Yet, despite universal healthcare coverage and a universal response coordinated by the Ministry of Health (MoH), a need for tailored approaches to population groups in the geographic and social periphery of Israel, is evident. One such group is that of religious Ultra-Orthodox Jewish populations. In some areas these groups have the highest infection rates due to a mix of risk factors, including low socio-economic status, crowded living conditions, minimal exposure to media platforms, and reliance on instructions from religious leaders who have not all conformed to governmental orders early on. Social distancing is anathema to the Orthodox Jewish communal way of life, and the government action of closing places of worship and study for the first time in the country’s history led to some early on opposition. Efforts to address COVID-19 among some of the ultra-religious populations require not only culturally tailored messaging but also culturally appropriate designated care facilities that can address religious needs such as dietary restrictions. Although a late response, the MoH together with other government authorities have invested great efforts to create the appropriate messaging as well as convene religious leaders and recruit their support by presenting data on contagion in synagogues for example. A recent commentary by Waitzberg and colleagues further details the unique needs of Ultra-Orthodox Jewish populations in Israel and the response to meet those needs [57].

A unique approach is also required in order to meet the needs of people from Arab minority groups (constituting about 20% of the Israeli population). Main public health communication efforts have been slow in providing linguistically and culturally tailored messages addressing unique cultural phenomenon such as lack of taking tests due to the perception, by some, that contracting the virus is shameful. In Israel’s northern periphery, of which the Arab speaking communities constitute 54% of the population, efforts to educate and spread culturally tailored messaging have been led through a community-based coalition of municipalities and NGOs. These include creating a central emergency response center for the Arab sector, creating a linked-up network of health and social welfare platforms, and recruiting respected Arab healthcare professionals to assist in educating the public. The response center together with MoH and health funds, are increasing their educational efforts with the aim of preventing an outbreak among the Muslim communities with the current month of Ramadan. An additional consideration during COVID-19 in the Arab population is the markedly high prevalence of chronic morbidity, especially those which are already proven risk factors for serious COVID-19 morbidity such as diabetes, smoking, chronic lung disease, and autoimmune disease. This will require a response to assist the overstretched and challenged healthcare providers in their ability to provide care and support and presage an impending crisis of backlog neglected non-COVID-19 disease and social support.

Other unique populations requiring specific consideration are those of non-citizens (mainly refugees and foreign workers), totaling about 250,000 people (~ 3% of the population in Israel). Non-citizens are not covered under the NHI law and therefore face significant barriers in access and provision of medical care (with the exception of some coverage to non-citizen children). These include financial, transportation, linguistic and cultural barriers, as well as fear of utilization of formal services due to suspicion of authorities. As part of the response to COVID-19, the Israeli MoH put together a team of representatives from non-citizen representative organizations and NGOs caring for non-citizens and issued clear instructions to all health funds and hospitals regarding the requirement to provide non-citizens COVID-19 tests and treatments at no point-of-care cost and without involving immigration officials. A COVID-19 test station was opened at a south Tel Aviv location, a concentrated living area of non-citizens. NGOs have also begun translating and distributing MoH COVID-19 messages in several languages, including Tigrinya and Thai.

Finally, among the many types of unique populations suffering from the larger societal effects of COVID-19, in Israel and worldwide, are the societally marginalized people in prostitution. Amongst other restrictions, the current lockdown measures in Israel also included closure of two mobile clinics that since 2008 have been treating people in prostitution and providing specific medical and social responses regarding sexual health, sexually transmitted diseases and gynecological care. As prostitution in Israel is illegal, brothels operate under the radar and therefore are also not part of the awareness of COVID-19 transmission detection efforts, leaving the vulnerable sex worker populations at an even greater risk.

### COVID-19 health equity perspectives: Australia

#### Elizabeth Sturgiss, John Furler

During non-pandemic times, there are many drawbacks to living on an island that is remote from many of the major cities in the world. However, a combination of our remoteness, early border closures, and strict distancing measures, has meant that by mid-May Australia had only 6989 confirmed cases with 98 deaths [58]. Australia has also had high levels of testing per head of population with over 943,500 tests conducted (mid-May 2020) [58], with testing initially limited to travelers or contact with a confirmed case, but now anyone with respiratory symptoms or a fever of unknown cause is encouraged to be tested.

Australia has had universal healthcare since 1975 [59] so testing and treatment for coronavirus are available to all citizens and permanent residents. Alongside Medicare, Australia has a private health system that performs more than 50% of elective surgery cases. Temporary visa holders, including international students, are not covered by Medicare and must hold personal, private healthcare insurance. General practice delivers the majority of primary care and prior to the pandemic, practitioners have been able to charge a gap payment at the point of care. Despite having universal healthcare, the inverse care law [60] is evident in Australia as demonstrated in mental healthcare prior to the pandemic [61]. For the COVID-19 response, the private healthcare system has been paid more than $1 billion to provide facilities for quarantine and treatment [62].

Australia’s pandemic response has had a strong focus on access to ongoing comprehensive primary care through telehealth. The Federal government introduced new payments for consultations conducted by telephone or video consultation. The earliest package mandated no out of pocket cost to the patient, but later versions of the legislation have allowed clinicians to charge a gap payment to patients deemed “not vulnerable” to the virus. Incentive payments have also been introduced for general practitioners who do not charge gap payments for vulnerable patients. While telehealth is an essential part of Australia’s COVID response to protect the healthcare workforce and patients, some of the equity issues associated with telehealth [63] are magnified when patients are living in lockdown. Lack of privacy, overcrowding and homelessness, poor access to devices and high-speed internet, could mean that patients will not benefit equally from this new form of service provision. Monitoring of access across different socioeconomic areas will be important for assessing the equity of the telehealth response.

The effects of ongoing colonisation of Aboriginal Torres Strait Islander populations is reflected in the higher rates of chronic disease and increased likelihood of living in overcrowded houses. The unique vulnerability of Indigenous communities has been recognised [64] and Australia will deploy the first rapid SARS-CoV-2 PCR tests to Indigenous regional and remote communities. This will allow for evacuation and/or re-housing in the event of an outbreak.

Access to appropriate and quality housing is a significant equity issue in the Australian pandemic response. In recent years, there has been an increase in the numbers of people living in unstable housing situations due to rapidly increasing rental prices and low public housing stock [65]. The federal government declared a six-month moratorium on all evictions, but this is yet to be passed into legislation by most state governments [66]. State governments are also piloting homelessness initiatives including hotel accommodation and expanding crisis accommodation, but the numbers of people who can be accommodated are inadequate. It is recognised that people on the streets and in overcrowded accommodation will be disproportionately affected by an increase in coronavirus infections [67].

The unemployment rate in Australia will reach 10% due to the shutdown of businesses to control the spread of infection [61]. The federal government recently launched a six-month wage subsidy scheme – “JobKeeper” – to assist people who had been made redundant or lost casual hours due to the pandemic. The payments are given to businesses who are then required to keep the person employed and pass on the payment. Almost one million Australians have lost their job with young people over-represented. The arts and entertainment sector has reported that many of the workers in their industry will ineligible for JobKeeper payments. Those who are ineligible will be able to apply for unemployment benefits that have also been increased and people on benefits have also received a $750 cash payment. The increases in welfare payments are the largest seen in the history of Australia’s social support system and highlight the need for a universal basic income guarantee.

### COVID-19 health equity perspectives: Colombia

#### Manuela Villar Uribe

Colombia is a High Middle-Income country that has invested steadily in the development and reach of its healthcare system, currently reaching over 96% of its legal residents with a comprehensive health insurance system [68]. The system is divided into two interconnected subsystems for those with the ability to contribute a percentage of their income (the Contributory Regime) and those in the informal sector or with income below a poverty threshold, who do not pay contributions to their insurance (the Subsidized Regime). The insured, regardless of income, age, gender or geographic location within the country are entitled to nearly all health services they will require with the exception of technologies without proven efficacy and cosmetic surgeries. Services are coordinated by private or semi-private insurers who, with a per-capita allocation from a nationally pooled fund are required to purchase services from public or private health care providers. The system was designed to ensure the equitable distribution of health services across the population and evidence suggests that although there are great achievements, inequalities in access to and quality of care persist [68–70].

With a strong health system and a clear understanding of the COVID-19 circumstances as a both a health and an economic crisis, Colombia has embarked on innovative measures that aim to reduce the impact on inequality in the country. After initiating a national-level lockdown in the last week of March, the National Government began to implement key measures to reduce the economic impact on lower income and minority populations by distributing boxes of food supplies, targeting an income payment/subsidy of approximately US$20 per family, increasing the number of beneficiaries in the conditional cash transfer program and facilitating tax returns for small companies and self-employed tax payers. Along with the efforts to increase availability of hospital beds and testing facilities, the health sector has implemented innovative ideas such as the provision of home-based care and telemedicine for higher-risk groups, with the aim of reducing their unnecessary contact with the health system, the development of task-shifting strategies to allow lower cadre health workers to provide basic but essential primary health care services [71], the rapid but streamlined facility licensing procedures for the use of non-hospital facilities for care provision to ensure quality service delivery, bonus payments for healthcare workers and importantly, a cash payment to low income persons infected by COVID-19 [72].

It is too early to know what the impact of the economic/social and health sector policies will have on inequalities in Colombia, however, a few factors that have not been directly addressed by recent policies could prove to be continuing challenges. A first major challenge is the over 1 million poor Venezuelan migrants that have entered the country in the past years of which a large percentage have no access to formal ambulatory health services or insurance protection [73]; these migrants are likely to suffer from a greater health and economic impact of these crises. A second factor is the large number of (more than 120,000) and levels of crowding of imprisoned population in Colombia (more than 50%), a remnant of the recently ended war [74], that creates great vulnerability to contracting the virus for this population. A third factor is the unaddressed inequalities in health service provision that have become endemic to the health system; inequalities in access across different wealth quintiles, inequalities in quality of care and inequalities in the realization of health care rights [68–70].

### COVID-19 health equity perspectives: Belgium

#### Sara Willems

Belgium has a fee-for-service health care system with a complementary patient list system at the level of primary care (67% of all Belgians is inscribed on a patient list), free choice of GP and direct access to secondary care. Primary health care plays an important role in the detection and the initial care of patients suspected of COVID-19. Patients with respiratory problems and/or fever are asked to contact their GP by phone. He/she does an intake interview and either reassures the patient when no COVID infection is suspected or gives advice on how to deal with the disease from home when a mild infection is suspected. Patients with more severe complaints are referred to a community-based triage point where general practitioners on-duty perform a physical examination and do technical investigations if the equipment to do so is provided in the triage facility: PO2 (always possible), CRP measurement (usually possible), RX thorax (rarely possible). Patients who need more intense treatment are send to hospital, following a region-wide triage and referral protocol. Patients recovering at home are followed up by their GP trough online and/or phone consultations.

Introducing this two-step primary care based approach allows not only for an efficient use of health care resources by decreasing importantly the burden of unnecessary use of the hospital emergency departments. It also reduces inequity in the detection of COVID patients, the initial care and the follow-up of the positive patients. Hence, all patients have access to the GP, to the triage centre and to the hospital. However, despite this universal accessible health care system some vulnerable groups experience major problems. First, the government communication on the measurements taken including this two-step procedure does not or hardly reach some vulnerable population groups: people not speaking one of the native languages, people with mental disorders or with limited health literacy, and homeless patients. Information is often focused on the white middle class and is too complex or does not take into account the difficult social circumstances in which some of these patients live. Because of lacking information in other languages than the national languages, some patients with a migration background turn to the available information from their home country resulting sometimes in behavioral patterns not conform the national guidelines regarding e.g. social distance but also health care use, making them vulnerable for social blaming. Some initiatives were therefore taken to translate health promotion materials and many practices actively reach out to vulnerable patients or invest in active follow-up procedures. This often time-consuming work is not remunerated in the fee-for-service system. Secondly, some patients, such as many of the homeless patients, do not have a fixed GP and experience therefor problems in accessing the health care system since direct access to the hospital is discouraged. Thirdly, although health care personnel is doing everything they can to provide health care to all who need it, it is unclear what the longterm financial consequences will be of hospitalization. Many patients, especially those without a voluntary hospitalization insurance and those who became jobless, fear personal bankruptcy. Even in a high income country with a strong welfare system such as Belgium the impact of becoming a COVID-19 patient might therefor largely contribute to inequity in health. Additionally, this gap might also be aggravated by the social differences in access to education (having access to computer at home to follow lessons …), in the ability to find a new job in a difficult economic context, and in the resilience to deal with multiple problems following the COVID pandemic on top of the already difficult social context in which many of our vulnerable population groups live.

## Data Availability

Not applicable.
